# Genomic Analysis of the Endangered Fonni’s Dog Breed: A Comparison of Genomic and Phenotypic Evaluation Scores

**DOI:** 10.3390/ani13050818

**Published:** 2023-02-23

**Authors:** Matteo Cortellari, Arianna Bionda, Raffaella Cocco, Sara Sechi, Luigi Liotta, Paola Crepaldi

**Affiliations:** 1 Department of Agricultural and Environmental Sciences, Milan University, Via Celoria, 2, 20133 Milan, Italy; 2 Department of Veterinary Medicine, Sassari University, Via Vienna, 2, 07100 Sassari, Italy; 3 Department of Veterinary Sciences, Messina University, Viale Palatucci, 13, 98168 Messina, Italy

**Keywords:** Italian dog breed, SNPs, dog breed conservation

## Abstract

**Simple Summary:**

The Fonni’s dog is a local breed selected for livestock and property guarding by Sardinian farmers since ancient times; however, this breed is now at high risk of being lost. The present study analyses the genomic background of 30 Fonni’s dogs genotyped with a high-density SNPchip and compares the genomic score obtained by admixture analyses with two morphological scores based on breed typicality and official judges’ evaluation. Genomic analyses showed that this breed is close to other shepherd dogs, but at the same time presents a unique genetic signature. All three scores were positively correlated, with higher values between the genomic and typicality scores. The judges’ score of the included dogs was a little variable and, therefore, could be improved to better rank dogs and include features that are particular to the breed. A shared vision between breeders and the Italian kennel club, as well as the support of dedicated regional programs, are fundamental in order to recover and preserve the Fonni’s dog.

**Abstract:**

The Fonni’s dog is an ancient Sardinian breed for livestock and property guarding. In recent years, the number of new registrations to the breeding book has slumped and, thus, this breed risks being lost forever. This work refocuses attention to the Fonni’s dog, analysing its genomic makeup and comparing different phenotypical and genetic evaluation scores. Thirty Fonni’s dogs were ranked by their general accordance to the breed typicality (typicality score) and to the provisional standard by official judges (judges’ score). They were genotyped with a 230K SNP BeadChip and compared with 379 dogs of 24 breeds. Genomically, the Fonni’s dogs placed themselves near shepherd dogs and showed a unique genetic signature, which was used to create the genomic score. This score better correlated with typicality (ρ = 0.69, *p* < 0.0001) than the judges’ score (ρ = 0.63, *p* = 0.0004), which showed little variability among the included dogs. Hair texture or colour were significantly associated in the three scores. The Fonni’s dog is confirmed as a well-distinguished breed, despite being selected mainly for its work abilities. The evaluation criteria used during dog expositions can be improved to increase their variability and include elements typical of the breed. The recovery of the Fonni’s dog would be possible only with a shared vision between the Italian kennel club and breeders, and the support of regional programs.

## 1. Introduction

The Fonni’s dog is an autochthonous Italian dog breed that originated in Sardinia in ancient times. Many testimonies come from XIX-century authors [1,2,3,4,5]. All of them described peculiar local dogs used as property and livestock guardians, but also for hunting hares and boars. They accented their absolute loyalty to their owner, contrasting with their fierce hostility towards strangers, as well as their strength, speed, and good health. The appearance of these dogs was considered unpleasant and coarse, even grim, due to their surly eyes and sharp fangs.

However, the origins of Fonni’s dogs are much more ancient [6]. It is believed that they were created through the cross between sighthounds and molossers [2,3,5,7]. This hypothesis has been recently supported by a genomic study that revealed that coursing hounds such as Salukis or Pharaoh hounds, as well as molossoid livestock guardians such as Komondors or Neapolitan mastiffs, are plausible ancestors of the Fonni’s dog and probably arrived in Sardinia following the peoples from the eastern Mediterranean and North African regions who then populated the isle [8]. Certainly, a large and tail-less dog was already present in Sardinia before the advent of the Romans, but the most ancient evidence of the presence of dogs resembling the modern Fonni’s dogs dates back to the Bronze Age (XIX century B.C.–II century A.D.) [6,7].

The selection of the Fonni’s dog has been long based solely on their aptitude for work and behavioural characteristics: they have been long used as livestock and property guardians, which enhanced their strong protectiveness towards their territory and owners. On the other hand, since an appearance-based selection has never been a priority, to this day, this breed shows considerable morphologic variability [6], although some commonalities can be found, such as the characteristic “monkey face”: the oval amber eyes, set in front and close one to each other, together with strongly developed eyebrows, conferring to these dogs a very intense, sad, and authoritative expression. The coat is usually double, with a course outer coat (defined as “goat” hair) and a woolly undercoat, but a short-haired variety exists. Moreover, some dogs are born with a natural bobtail [6,9].

What is remarkable is that, despite this variability and the absence of a strong selection toward a defined standard, several authors reported that, from a genetic perspective, the Fonni’s dog meets all the requirements to be considered a full-fledged breed [6,8,10]. It is likely that both a certain degree of geographical isolation and the incredible support of the Fonni’s dogs to the prosperity of early Sardinian people contributed to the conservation of this breed through the centuries. At present, the Fonni’s dog is in the process of being recognised as a breed; in 2013, it obtained, by the National Agency of the Italian kennel club (ENCI), a specific breeding book (Open Additional Register, RSA) aiming to conserve endangered local dog populations, and a provisional breed standard has been deposited [9]. However, ENCI’s data show that, while during the first years of RSA, about 90 dogs—including both founders, enrolled if judged as compelling with the standard by an expertise committee, and their offspring—were annually registered, in the last few years, the breed has seen a slump in registrations to the breeding book, reaching the dramatically low number of 16 subjects enrolled in 2020 (www.enci.it/libro-genealogico/razze/cane-fonnese, accessed on 20 December 2022). At least in part, this was probably due to the disappointment of breeders, who do not feel adequately represented by the morphometric preferences and the subjects’ evaluations proposed by ENCI. In light of the above, it is of paramount importance to bring the attention back to the Fonni’s dog breed, which otherwise would seriously risk being lost forever, despite the efforts of ENCI.

Today, we dispose several genomic tools that can be of help in the management of animal populations; genetics has long been applied to livestock selection, but its use is still limited in pet breeding. However, especially when we consider small, local breeds, the implementation of a genomic evaluation would contribute to their conservation; not only it can be used to choose the best mating schemes and preserve genetic diversity, but it could accompany the traditional phenotypical judgement to identify the most idoneous breeding dogs. 

Therefore, the aims of the present study are (i) to investigate the genomic makeup of the Fonni’s dog with respect to other breeds; (ii) to compare three different evaluation scores, both phenotypical and genetic; and (iii) to identify genomic regions differentiating Fonni’s dogs with different physical features.

## 2. Materials and Methods

### 2.1. Phenotypical Evaluation

In the present study, 30 Fonni’s dogs were photographed and judged by breed experts. Enrolled dogs were born between 2007 and 2018. Data were collected about hair colour and texture (goat, woolly, or short), eye colour, tail presence, and type of bite (scissor, pincer, overshot, or undershot). Head (muzzle length, muzzle width, and stop angle), limbs (shoulder and leg angles, and anterior and posterior stance), chest (topline, length, and depth of chest), coat texture, size, height at withers, bone, responsiveness, and predatory behaviour were evaluated using a 20-point rating scale, where 10 corresponded to a perfect accordance with the standard [9]. Each dog was assigned a score according to the % deviation from the perfection (‘judges’ score’).

Moreover, a local researcher with a deep knowledge of the breed ranked the Fonni’s dogs according to their general appearance and compliance with the breed typicity (0–100 points ‘typicality score’). In this case, the subjects were evaluated as a whole, with particular emphasis given to the typical traits of the breed. The so-called “monkey face” represents a very peculiar element; therefore, great importance was given to the head and its proportions, as well as eye colour, shape, and position. Among the other elements carefully evaluated were also the coat texture and colour. 

**Figure 1:**
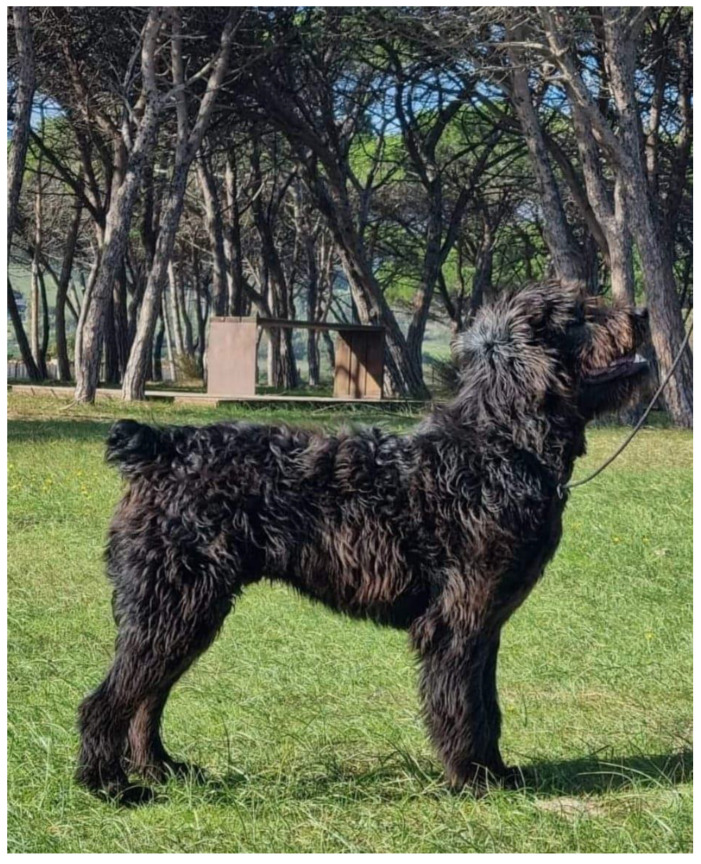
An example of brachyure Fonni’s dog.

An example of a Fonni’s dog is reported in Figure 1.

### 2.2. Genomic Study

Blood samples of the aforementioned 23 unrelated Fonni’s dogs were collected in accordance with the Ethics Committee’s statement of the University of Messina, number 040/2020bis. DNA was extracted using DNeasy Blood and Tissue Kit (QIAGEN^®^, Hilden, Germany) following the manufacturer’s instructions and then genotyped via outsourcing with Canine 230K SNP BeadChips (Illumina^®^, San Diego, CA, USA).

This dataset was merged with publicly available SNP chip data [10,11,12] of 7 Fonni’s dogs and 379 dogs from 24 Italian and foreign breeds that could be historically or phenotypically related to Fonni’s dogs and have been indicated by Sardinian veterinarians as the most diffuse in Sardinia, especially for hunting activities: Pastore Apuano (APUA, n = 19), Bergamasco Shepherd (BERG, n = 15), Bracco Italiano (BRAC, n = 12), Cane Corso (CCIT, n = 22), Cirneco dell’Etna (CIRN, n = 24), Pastore d’Oropa (DORO, n = 15), English Setter (ESET, n = 15), German Shepherd dog (GSD, n = 10), German Shorthaired Pointer (GSHP, n = 10), German Wirehaired Pointer (GWHP, n = 2), Italian Greyhound (IGIT, n = 20), Irish Setter (ISET, n = 9), Lagotto Romagnolo (LAGO, n = 24), Lupino del Gigante (LUGI, n = 23), Lupo Italiano (LUPO, n = 24), Mannara’s dog (MANN, n = 12), Maremma and the Abruzzi Sheepdog (MARM, n = 20), Mastiff (MAST, n = 10), Neapolitan Mastiff (NMIT, n = 12), Pastore della Lessinia e del Lagorai (PALA, n = 10), Pastore della Sila (SILA, n = 14), Segugio Italiano a Pelo Forte (SIPF, n = 16), Segugio Italiano a Pelo Raso (SIPR, n = 16), and Spinone Italiano (SPIN, n = 24).

A quality control was performed on the genomic data using PLINK 1.9 [13]: only dogs with missingness per individual < 0.1 and SNPs with missingness per marker < 0.05 and minor allele frequency > 0.01 were retained. Related dogs were excluded. 

The genetic structure of the selected populations was represented with a multidimensional scaling (MDS) analysis, performed with PLINK 1.9. Using ADMIXTURE 1.3 [14], the individual genetic admixture was investigated for a number of clusters (K) ranging from 2 to 28. For each dog, the probability of being assigned to the different clusters (Q-score) was calculated. The best-fitting model was identified as the one with the lowest cross-validation value (cv-value); according with this model’s Q-score of their own cluster, a ‘genomic score’ was attributed to all the Fonni’s dogs.

The studied Fonni’s dogs were grouped according to the following phenotypical characteristics: (i) Type of bite (n. 24 scissor bite vs. n. 5 pincer bite); (ii) Tail presence (n. 18 brachyure vs. n. 12 tailed); (iii) Hair length (n. 25 longhaired vs. n. 5 shorthaired); and (iv) Hair texture (n. 17 “goat” hair vs. n. 8 woolly hair). Each pair of groups was compared using two genomic analyses: the Wright’s fixation index (F_ST_), using PLINK 1.9, and the single SNP cross-population extended haplotype homozygosity (XP-EHH), using SELSCAN 1.1.0 [15]. These analyses identify genomic regions differentiating two groups of individuals according to the amount of differentiation in their allele frequency [16,17] and the haplotype lengths at each marker [18,19], respectively. The SNPs ranking in the top 1% of the empirical distribution of both the analyses were considered relevant and mapped to the reference genome assembly CanFam3.1. The genes containing these relevant markers were further investigated.

### 2.3. Statistical Analysis

Statistical analyses were performed using JMP^®^ 15 (SAS Institute Inc., Cary, NC, USA). Descriptive statistics were generated. Pearson correlation coefficient (ρ) was calculated between the three different scores (genomic, judges’, and typicality scores). One-way ANOVA and Chi-squared test were used to compare continuous and categorical variables in different groups, respectively. *p*-values were considered significant if <0.05.

## 3. Results

### 3.1. Genomic Analysis

The dataset obtained after the quality check and the exclusion of related subjects consisted of 120853 SNPs and 378 dogs: 30 Fonni’s dogs, 18 Pastore Apuano (APUA), 11 Bergamasco shepherds (BERG), 12 Bracco Italiano (BRAC), 20 Cane Corso Italiano (CCIT), 21 Cirneco dell’Etna (CIRN), 15 Pastore D’Oropa (DORO), 15 English Setters (ESET), 10 German Shepherd dogs (GSD), 10 German Shorthaired Pointers (GSHP), 2 German Wirehaired Pointers (GWHP), 14 Italian Greyhounds (IGIT), 9 Irish Setters (ISET), 23 Lagotto Romagnolo (LAGO), 18 Lupino del Gigante (LUGI), 23 Lupo Italiano (LUPO), 12 Mannara dogs (MANN), 16 Maremma and the Abruzzi Sheepdogs (MARM), 10 Mastiffs (MAST), 10 Neapolitan Mastiffs (NMIT), 10 Pastore della Lessinia e del Lagorai (PALA), 14 Pastore della Sila (SILA), 16 Segugio Italiano a Pelo Forte (SIPF), 16 Segugio Italiano a Pelo Raso (SIPR), and 23 Spinone Italiano (SPIN). 

**Figure 2:**
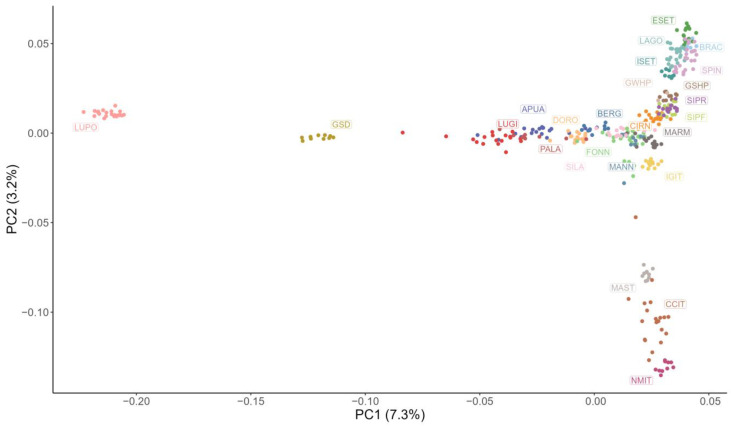
Multidimensional scaling analysis plot showing the first two principal components (PC). Each point represents a subject and each colour a breed: Fonni’s dog (FONN), Pastore Apuano (APUA), Bergamasco Shepherd (BERG), Bracco Italiano (BRAC), Cane Corso Italiano (CCIT), Cirneco dell’Etna (CIRN), Pastore D’Oropa (DORO), English Setter (ESET), German Shepherd dog (GSD), German Shorthaired Pointer (GSHP), German Wirehaired Pointer (GWHP), Italian Greyhound (IGIT), Irish Setter (ISET), Lagotto Romagnolo (LAGO), Lupino del Gigante (LUGI), Lupo Italiano (LUPO), Mannara dog (MANN), Maremma and the Abruzzi Sheepdog (MARM), Mastiffs (MAST), Neapolitan Mastiff (NMIT), Pastore della Lessinia e del Lagorai (PALA), Pastore della Sila (SILA), Segugio Italiano a Pelo Forte (SIPF), Segugio Italiano a Pelo Raso (SIPR), and Spinone Italiano (SPIN).

A representation of the first two components of the MDS analysis is shown in Figure 2. In this plot, LUPO and GSD are both isolated from the other breeds; along the vertical axis, the molossers (MAST, CCIT, and NMIT) are well identifiable and are located close together, far from the other breeds, as well as the hunting dogs (ESET, BRAC, SPIN, ISET, GSHP, GWHP, SIPR, SIPF, and CIRN). Regarding shepherd dogs, a partial differentiation between livestock guardians (SILA, MANN, MARM, and Fonni’s dogs) and herding dogs (LUGI, PALA, APUA, DORO, and BERG) is appreciable. 

**Figure 3:**
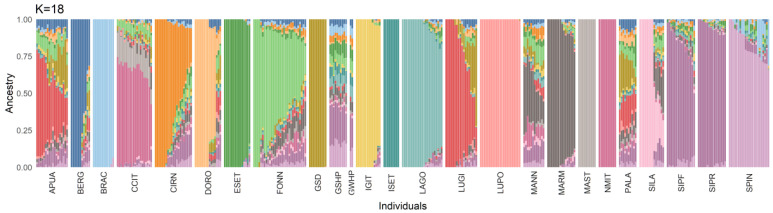
Admixture analysis for the best-fitting model. Each bar represents the genetic composition of a subject, according to the Q-value of the 18 clusters (K) included. Breeds included: Fonni’s dog (FONN), Pastore Apuano (APUA), Bergamasco Shepherd (BERG), Bracco Italiano (BRAC), Cane Corso Italiano (CCIT), Cirneco dell’Etna (CIRN), Pastore D’Oropa (DORO), English Setter (ESET), German Shepherd dog (GSD), German Shorthaired Pointer (GSHP), German Wirehaired Pointer (GWHP), Italian Greyhound (IGIT), Irish Setter (ISET), Lagotto Romagnolo (LAGO), Lupino del Gigante (LUGI), Lupo Italiano (LUPO), Mannara dog (MANN), Maremma and the Abruzzi Sheepdog (MARM), Mastiffs (MAST), Neapolitan Mastiff (NMIT), Pastore della Lessinia e del Lagorai (PALA), Pastore della Sila (SILA), Segugio Italiano a Pelo Forte (SIPF), Segugio Italiano a Pelo Raso (SIPR), and Spinone Italiano (SPIN).

At K = 2, the admixture analysis separated LUPO from all the other breeds; at K = 3, the molossers could be identified by a common signature; then, all the following clusters identified single breeds, one at a time. The best-fitting admixture model was identified at K = 18 (cv-value = 0.58, Figure 3). In this model, BRAC, ESET, GSD, IGIT, ISET, LAGO, LUPO, MAST, and NMIT were the breeds displaying the least introgression. Fonni’s dog showed a unique genetic signature and the dogs included in this study had, on average (±SD), a Q-score related to their own cluster of 61.7 ± 22.0%, ranging from 19.2 to 100%. In particular, seven subjects were comprised in the fourth quartile (>75%), eleven in the third (50–75%), ten in the second (25–50%, but eight dogs were over 40%), and one in the first (<25%, Table S1). The second most represented clusters, accounting for at least 5% of the Fonni’s dogs’ genomic background, were: MARM (n = 12, 10.7 ± 0.03%), SIPF/SIPR (n = 5, 9.5 ± 0.03%), NMIT/CCIT (n = 2, 13.4 ± 0.01%), and MAST (n = 1, 9.8%). MARM was also highly represented in the background of other shepherd dogs: MANN, PALA, and SILA. 

The results of the comparison between groups of Fonni’s dogs with different phenotypical characteristics are shown in Table 1. In particular, 23 different genes harboured SNPs in the top 1% of the distribution of both F_ST_ (0.16–0.46) and XP-EHH (2.7–4.9) analyses comparing scissor bite and pincer bite groups. Similarly, 21 genes differentiated brachyure and tailed dogs (F_ST_: 0.10–0.39; XP-EHH: 2.7–4.6); 23 genes differentiated longhaired and shorthaired dogs (F_ST_: 0.22–0.53; XP-EHH: 2.9–6.6); and 13 genes differentiated dogs with “goat” hair and woolly hair (F_ST_: 0.15–0.52; XP-EHH: 2.8–5.8).

### 3.2. Phenotypic Description

Of the 30 studied Fonni’s dogs, 12 (40%) had a normal tail and 18 (60%) were brachyure. The majority of dogs had a scissor bite (80%) or a pincer bite (17%), both accepted by the breed standard; only one dog (0.3%) had an overshot bite.

Grey, in different shades, was the most represented coat colour (50%), followed by brindled (27%) and black (17%); only one subject was found for honey and white colours. Moreover, 63% of dogs did not display white spotting. Most of the dogs had the typical “goat” hair (57%), whereas in eight, the coat was considered “woolly” (27%); the short-coated variety was also well represented (17%).

The phenotypic evaluation performed by breed experts was available for 29 out of the 30 studied dogs. Many of the evaluated parameters showed small variability: mean (±SD) coefficient of variation (CV) was 6.27 ± 3.98%. One parameter (shoulder angle) had a CV = 0; the parameter with the greatest variability was hair texture (CV = 14.1%). On average (±SD), the judges’ score was of 97.6 ± 3.1%; 11 dogs (41%) were evaluated as perfectly in standard (0 faults) and only two dogs had a relatively low score, equal to 86.3 and 91.3%. Individual data about the phenotypical characterisation and the three scores are reported in Table S1.

We investigated the correlation between the different scores (genomic, judges’, and typicality): the genomic score significantly correlated with both typicality (ρ = 0.69, *p* < 0.0001) and judges’ score (ρ = 0.63, *p* = 0.0004); the two phenotypic scores were less, but still significantly, correlated (ρ = 0.45, *p* = 0.02). 

Statistical analyses did not show any significant association between the genomic or the typicality score and white spotting, tail (tailed or brachyure), and bite. On the other hand, a significant association was found in the hair texture category (short, woolly, or goat), with higher values of Q-score (*p* = 0.005) and typicality (*p* < 0.0001) for goat-haired dogs, and a worse typicality score for short-haired ones. Typicality score only was significantly associated with coat colour (*p* = 0.007), with a preference for ash grey, black, and brindled honey dogs and lower values for brindled dogs, and regarding eye colour (*p* = 0.01), amber was the preferred colour and black was the least favourite. 

## 4. Discussion

Fonni’s dogs have long been bred in Sardinia for guarding purposes. However, the recent confrontations between breeders and ENCI contributed to the fall in the number of registrations in the breeding book, putting at risk the survival of this breed. In this work, we aimed to renew the attention to this population, also comparing different morphological and genomic scores and indicating possible tools that might help manage local canine breeds.

From a genomic perspective, according to the analyses of the Fonni’s dog’s population structure and genetic background, this breed appears very distinct from other Italian and foreign populations, as already recorded in Talenti et al. (2018) [10], where 185 breeds were investigated. As expected, the Fonni’s dog shares similarities with other livestock guardian dogs, in particular with the Maremma and the Abruzzi sheepdog, which is the most diffuse livestock guardian. Observing in detail the background of the single subjects, we noticed that there is a remarkable degree of variability in the presence of a genetic signature attributable to the Fonni’s dog breed. In this regard, these genomic tools might be of great help in the evaluation of these dogs, particularly of those that do not perfectly adhere to the morphological standard, and, thus, in the selection of the founders to register in the breeding book.

Comparing this genomic score with that used by ENCI judges during the expositions and a typicality score based on the general appearance of the individuals, we found a strong and positive correlation between all of them, but with the lowest coefficient between the two morphological scores. Generally speaking, the dogs that showed a very high genomic score also presented a high typicality score, but with some exceptions: this could indicate that, despite the presence of clear typical features, a phenotypical variability is still present in the breed. We propose that the dogs showing the highest genomic scores might be subjected to an accurate phenotypical evaluation to identify what the elements that best define them are, always considering both historical documents and the possible preferences of the single breeders, which are reflected in their dogs. With regard to the judges’ score, we found that only a very small number of parameters, such as the hair texture, shows variability among the enrolled dogs, whereas the majority were evaluated as perfect for almost all of them. This, together with the fact that the genomic score better correlated with the typicality score rather than the judges’ one, could suggest that including other criteria might be advantageous. For example, some typical features of the breed were not evaluated (e.g., presence or absence of the tail) or indicated as open answer (e.g., eye colour), making it difficult to collect data about them in the population. The breed, indeed, shows some peculiar traits, such as frontal amber eyes, hostile expression, and goat hair texture, that should be carefully reported in the evaluations in order to understand their variability in the population and considered in the decision to register a dog into the breeding book. Furthermore, since the selection of these dogs has historically been based on their behaviour and performance in work, it would be appropriate to also include these elements in their evaluation, in order not to lose the guarding attitude of this breed. For example, distrust towards strangers and territoriality are necessary characteristics for guard dogs, and therefore are traits to be rewarded.

The second part of our study focused on the identification of selection signatures of Fonni’s dogs with different phenotypes, including the bite, the presence of tail, and the hair length and texture. From the comparison of longhaired and shorthaired subjects, the latter composing 17% of the sample, we identified *RSPO2*, a gene known to be responsible for the presence of furnishing and wire hair in the canine species [20]. Moreover, the evidence of different combination of mutations in *RSPO2* and *FGF5* associated with the longhair phenotype in several dog breeds suggests an epistatic interaction between the two and, thus, that *RSPO2* plays a role in the determination of the hair length in this species [20,21,22]. *CREM*, another gene we identified, is involved in the regulation of *AP-1* [23], a dimer of *FOS* and *JUN* that seems to regulate the composition of dog undercoat [24]. In addition, a recent study involving over 20000 dogs reported an association between fur texture and *EXT1* and *ANGPT1* genes [25]. Consistently, mice harbouring mutations in *EXT1* were characterised by densely distributed hair follicles which only presented anagen phase, as well as accelerated hair regrowth [26]. Interestingly, two of the identified genes are, instead, associated with coat colour: *USH2A* is related to roan and ticked colour in dogs [27], whereas *LYST* is known to determine beige, aleutian, and diluted colour in mice, minks, and bovines, respectively [28,29,30], and also to cause Chediak–Higashi syndrome in several species [28,31,32].

Among the Fonni’s dogs enrolled in the present study, 60% presented a short tail, a feature that is accepted by the provisional breed standard. This phenotype in many, but not all, dog breeds has been associated to a mutation in the *T* gene, which is homozygous lethal [33,34]. The comparison of brachyure and normal-tailed Fonni’s dogs did not identify it, but the genes that differentiated these two groups included *DISC1*. This gene acts in the neuroectoderm, regulating somite and tail formation, but its main role regards brain formation. A study on zebrafish showed that *DISC1* mutations can also lead to the development of an abnormally shaped and truncated tail [35,36].

Lastly, we compared Fonni’s dogs presenting the two accepted bites: scissor and pincer (17%). Interestingly, we found the *COL13A1* gene, which has been shown to regulate bone growth and resorption. An overexpression of *COL13A1* can abnormally increase bone formation and, therefore, it is supposed to influence bone modelling, in particular in relation with its mechanical use [37]. The same study also highlighted a probable interaction between *COL13A1*, *IGF-II*, and *RUNX2*, the latter being one of the candidate genes accounting for the differences in muzzle length in dogs [38,39] and class II malocclusions in humans [40]. 

## 5. Conclusions

Our study focused on a local dog breed that, due to management reasons, might be lost in the near future. Indeed, the zootechnical management of the Fonni’s dog population, like several other breeds with limited diffusion, sees “livestock keepers” choosing the breeding dogs through a morphological and functional evaluation, often driven by their experience or that of their family. In this context, it frequently occurs that the selected dogs deviate from the morphological criteria of the provisional breed standard provided by ENCI. As a consequence, litters are not regularly registered and cannot obtain a pedigree, not contributing to the official breed statistics. To limit this problem, it is of great importance to solve the disagreements between breeders and the kennel club, for example, improving the role and the responsibility of the breed club and the livestock keepers, which actually deal with the preservation activities over time, and creating accurate mating plans and exchanging of dogs between breeders that share a common vision of the breed. Another suggestion might be to follow the successful example of other breed clubs, including the Mannara dog breed club [41], establishing a roving commission of ENCI judges who personally visit the farms of the rural areas where the possible founder dogs live, giving the opportunity to increase the total and breeding population size. This direct contact with the breeders would also allow them to collect their concerns about the management and vision of the breed, and thus improve the breed standard like it was a “participatory project”, it being, in fact, a dynamic document that should evolve accordingly with the population. 

Moreover, we propose to implement the use of genomics, which demonstrated to be useful in avoiding excessive inbreeding and in distinguishing the subjects that, from a genomic perspective, should be highly representative of the breed. To leverage these tools, the development of a reference dataset including updated data from the highest number of breeds possible is relevant, thus filling the gaps recorded for the small Italian local populations, such as Fonni’s dogs. In our opinion, this would pave a way toward more informed and ethical dog breeding.

The process of recovery and conservation of local breeds certainly demands great efforts and time, especially in the peculiar pastoral reality of southern Italy and isles. However, letting a population die out would also mean losing the related history, tradition, and biodiversity, which is not an acceptable option.

## Figures and Tables

**Table 1:** Genes identified by both F_ST_ and XP-EHH analyses comparing Fonni’s dogs with different phenotypes.

Compared Groups	Gene Name	CFA	Start	End	Complete Name
Scissor bite (*n* = 24) vs. Pincer bite (*n* = 5)	*PLXDC2*	2	12609638	13056951	Plexin domain-containing 2
	*COL13A1*	4	20838674	20985200	Collagen type XIII alpha 1 chain
	*TYSND1*	4	21106873	21132547	Trypsin domain-containing 1
	*PPA1*	4	21132550	21181653	Pyrophosphatase (inorganic) 1
	*EIF4EBP2*	4	21338907	21357830	Eukaryotic translation initiation factor 4E binding protein 2
	*ADAMTS14*	4	21534016	21612585	ADAM metallopeptidase with thrombospondin type 1 motif 14
	*NUDT13*	4	23695064	23711400	Nudix hydrolase 13
	*ECD*	4	23708872	23740243	Ecdysoneless cell cycle regulator
	*USP54*	4	24030697	24094273	Ubiquitin specific peptidase 54
	*DYNC2H1*	5	28387793	28727334	Dynein cytoplasmic 2 heavy chain 1
	*STX8*	5	33834271	34087011	Syntaxin 8
	*SLC9A2*	10	40338284	40560356	Solute carrier family 9 member A2
	*SEMA6A*	11	6116854	6177817	Semaphorin 6A
	*CCDC192*	11	16794360	16983758	Coiled-coil domain-containing 192
	*ALLC*	17	2197913	2215973	Allantoicase
	*SEMA3C*	18	20457610	20635275	Semaphorin 3C
	*EEFSEC*	20	2155781	2407022	Eukaryotic elongation factor, selenocysteine-trna-specific
	*DIAPH3*	22	15572575	16079207	Diaphanous-related formin 3
	*PTPRT*	24	30000993	30764482	Protein tyrosine phosphatase, receptor type T
	*XKR4*	29	6640126	7064029	XK-related 4
	*CTNND2*	34	2521662	3439858	Catenin delta 2
	*MARCH6*	34	3884154	3944135	Membrane-associated ring-CH-type finger 6
	*STK39*	36	12866818	13307782	Serine/threonine kinase 39
Brachyure (*n* = 18) vs. Tailed (*n* = 12)	*ADGB*	1	37752823	37915540	Androglobin
	*C19orf81*	1	106012709	106306177	Chromosome 19 open reading frame 81
	*SHANK1*	1	106086132	106128643	SH3 and multiple ankyrin repeat domains 1
	*NEBL*	2	11891943	12229221	Nebulette
	*DISC1*	4	7520420	7822558	Disrupted in schizophrenia 1
	*WAPL*	4	34219462	34536265	WAPL cohesin release factor
	*DOCK2*	4	41778325	42177874	Dedicator of cytokinesis 2
	*MAD1L1*	6	14888955	15236462	MAD1 mitotic arrest deficient like 1
	*NEGR1*	6	74164157	74634072	Neuronal growth regulator 1
	*LAMA3*	7	64405533	64655273	Laminin subunit alpha 3
	*LONRF1*	16	36217906	36257537	LON peptidase N-terminal domain and ring finger 1
	*NCKAP5*	19	35633416	36498816	NCK associated protein 5
	*ESD*	22	4548798	4571383	Esterase D
	*LRCH1*	22	4573064	4781189	Leucine rich repeats and calponin homology domain-containing 1
	*TRPM8*	25	45261107	45353285	Transient receptor potential cation channel subfamily M member 8
	*PITPNB*	26	21304452	21369641	Phosphatidylinositol transfer protein beta
	*B4GALT4*	33	22902375	22928826	Beta-1,4-galactosyltransferase 4
	*POGLUT1*	33	23122291	23152469	Protein O-glucosyltransferase 1
	*PARP14*	33	25763335	25801465	Poly(ADP-ribose) polymerase family member 14
	*FARS2*	35	5436316	5889563	Phenylalanyl-trna synthetase 2, mitochondrial
	*CCDC148*	36	4084608	4342485	Coiled-coil domain containing 148
Longhaired (*n* = 25) vs. Shorthaired (*n* = 5)	*GLIS3*	1	92278216	92724554	GLIS family zinc finger 3
	*CREM*	2	1751312	1828948	Camp-responsive element modulator
	*LYST*	4	4106784	4294142	Lysosomal trafficking regulator
	*PCNX2*	4	6341243	6699442	Pecanex homolog 2 (Drosophila)
	*HK1*	4	20449974	20519488	Hexokinase 1
	*TSPAN15*	4	20556860	20607961	Tetraspanin 15
	*COL13A1*	4	20838674	20985200	Collagen type XIII alpha 1 chain
	*SGPL1*	4	21666395	21718699	Sphingosine-1-phosphate lyase 1
	*MCU*	4	23309391	23511184	Mitochondrial calcium uniporter
	*ANXA11*	4	29250164	29266002	Annexin A11
	*SH2D4B*	4	29651821	29734014	SH2 domain-containing 4B
	*WAPL*	4	34219462	34536265	WAPL cohesin release factor
	*RGS6*	8	45791392	45975087	Regulator of G-protein signalling 6
	*OXR1*	13	7220958	7581210	Oxidation resistance 1
	*ABRA*	13	7587713	7599641	Actin binding Rho-activating protein
	*ANGPT1*	13	8068880	8312114	Angiopoietin 1
	*RSPO2*	13	8610233	8755897	R-spondin 2
	*EXT1*	13	17172851	17456469	Exostosin glycosyltransferase 1
	*HDAC9*	14	32788493	33304272	Histone deacetylase 9
	*KIAA1549*	16	9696592	9834339	KIAA1549
	*RAD54L2*	20	37966800	38084854	RAD54-like 2 (S. Cerevisiae)
	*WSCD2*	26	18574098	18691280	WSC domain-containing 2
	*USH2A*	38	11063439	11750372	Usherin
“Goat” hair (*n* = 17) vs. Woolly hair (*n* = 8)	*PHLPP1*	1	14027635	14233389	PH domain and leucine-rich repeat protein phosphatase 1
	*RHOJ*	8	37802907	37880272	Ras homolog family member J
	*PPP2R5E*	8	37947434	38085846	Protein phosphatase 2 regulatory subunit b’epsilon
	*SGPP1*	8	38193018	38228404	Sphingosine-1-phosphate phosphatase 1
	*GALNT16*	8	43099310	43187969	Polypeptide N-acetylgalactosaminyltransferase 16
	*SIPA1L1*	8	45110084	45260233	Signal-induced proliferation-associated 1 like 1
	*CHN2*	14	42217570	42513386	Chimerin 2
	*ZSWIM5*	15	15110474	15311001	Zinc finger SWIM-type containing 5
	*TMTC2*	15	24629162	25019828	Transmembrane and tetratricopeptide repeat-containing 2
	*C2CD3*	21	24127598	24407702	C2 calcium-dependent domain-containing 3
	*DNAJB13*	21	24314400	24327205	DnaJ heat shock protein family (Hsp40) member B13
	*PAAF1*	21	24338678	24381829	Proteasomal atpase-associated factor 1
	*B3GLCT*	25	8817641	8938884	Beta 3-glucosyltransferase

## Data Availability

Genomic data can be downloaded from the Gene Expression Omnibus database under accession number GSE121027 (https://www.ncbi.nlm.nih.gov/geo/query/acc.cgi?acc=GSE121027, accessed on 20 December 2022), GSE96736 (https://www.ncbi.nlm.nih.gov/geo/query/acc.cgi?acc=GSE96736, accessed on 20 December 2022), and GSE90441 (https://www.ncbi.nlm.nih.gov/geo/query/acc.cgi?acc=GSE90441, accessed on 20 December 2022).

## Supplementary Materials

The following supporting information can be downloaded at: https://www.mdpi.com/article/10.3390/ani13050818/s1, Table S1: Scores and description for all the enrolled Fonni’s dogs.
