# Supplementary material for: Genomic Analysis of the Endangered Fonni’s Dog Breed: A Comparison of Genomic and Phenotypic Evaluation Scores
Source: Animals (Basel). 2023 Feb 23;13(5):818. doi: 10.3390/ani13050818 (PMC10000202; doi:10.3390/ani13050818)
Supplement: Supplementary file 1 [file animals-13-00818-s001.zip › animals-2181270-supplementary.pdf]

Table S1. Scores and description for all the enrolled Fonni's dogs.

Genomic score	Typicality score	Judges' score	Sex	Coat colour	White spotting	Hair texture	Tail	Eye colour	Bite	Secondary breed
100%	100%	100%	Female	Gray (ash)	No	Goat	Tailed	Amber	Scissors	
100%	80%	100%	Female	Black	No	Goat	Brachyure	Amber	Scissors	
100%	70%	100%	Female	Black	No	Goat	Brachyure	Amber	Scissors	
97%	80%	100%	Male	Gray (ash)	No	Goat	Brachyure	Amber	Pincer	
86%	100%	99%	Female	Brindled honey	No	Goat	Brachyure	Amber	Scissors	
85%	90%	97%	Male	Gray	No	Goat	Brachyure	Dark amber	Scissors	
80%	100%	98%	Female	Brindled honey	No	Goat	Tailed	Amber	Pincer	
75%	80%	98%	Female	Gray (ash)	Yes	Goat	Brachyure	Amber	Pincer	
75%	60%	100%	Female	Gray (ash)	Yes	Goat	Brachyure	Amber	Scissors	
74%	70%	100%	Female	Gray (ash)	No	Goat	Brachyure	Amber	Scissors	
73%	30%	98%	Male	Brindled	Yes	Short	Tailed	Dark amber	Scissors	MARM
64%	60%	98%	Male	Gray (ash)	No	Goat	Tailed	Amber	Scissors	MARM
62%	60%	100%	Female	Gray (ash)	Yes	Goat	Tailed	Amber	Scissors	MARM
61%	60%	100%	Female	Gray (ash)	No	Goat	Brachyure	Amber	Scissors	SIPF/SIPR
58%	60%	95%	Male	Black	No	Woolly	Tailed	Amber	Scissors	SIPF/SIPR
58%	50%	98%	Female	Brindled	No	Short	Tailed	Amber	Scissors	MARM
57%	70%	100%	Male	Gray (dark)	Yes	Goat	Brachyure	Amber	Scissors	MARM
56%	40%	100%	Male	White	No	Woolly	Tailed	Dark amber	Scissors	MARM
54%	60%	97%	Female	Black	No	Woolly	Brachyure	Dark amber	Scissors	SIPF/SIPR
50%	90%	98%	Male	Gray (ash)	Yes	Woolly	Tailed	Dark amber	Scissors	SIPF/SIPR
48%	40%	99%	Female	Brindled	Yes	Short	Tailed	Dark amber	Scissors	MAST
45%	60%	94%	Male	Gray (light)	No	Woolly	Brachyure	Dark amber	Overshot	MARM
45%	40%	94%	Female	Honey	Yes	Woolly	Brachyure	Black	Scissors	MARM
44%	50%	97%	Male	Gray (ash)	No	Woolly	Brachyure	Amber	Pincer	NMIT/CCIT
43%	50%	98%	Female	Brindled honey	No	Goat	Brachyure	Amber	Pincer	NMIT/CCIT
43%	60%	99%	Male	Gray (ash)	Yes	Goat	Brachyure	Amber	Scissors	SIPF/SIPR
43%	40%	98%	Female	Gray (ash)	Yes	Woolly	Tailed	Amber	Scissors	MARM
30%	70%	91%	Male	Black	No	Goat	Brachyure	Amber	Scissors	MARM
28%	20%	86%	Female	Brindled	No	Short	Brachyure	Dark amber	Scissors	MARM
19%	10%		Female	Brindled	Yes	Short	Tailed	Black	Scissors	MARM

Secondary breed refers to the main cluster after the Fonni's dog's one (Q-value > 5%) identified in the admixture analysis: MARM = Maremma and the Abruzzi's sheepdog, SIPF/SIPR = cluster common to Segugio Italiano a Pelo Forte and Segugio Italiano a Pelo Raso; MAST = Mastiff.
